# Amphiphilic Triazine-Phosphorus Metallodendrons Possessing Anti-Cancer Stem Cell Activity

**DOI:** 10.3390/pharmaceutics14020393

**Published:** 2022-02-10

**Authors:** Evgeny K. Apartsin, Nadezhda Knauer, Ulf Dietrich Kahlert, Anne-Marie Caminade

**Affiliations:** 1 Laboratoire de Chimie de Coordination, CNRS, 205 Route de Narbonne, CEDEX 04, 31077 Toulouse, France; 2 LCC-CNRS, Université de Toulouse, CNRS, 31077 Toulouse, France; 3 Institute of Chemical Biology and Fundamental Medicine SB RAS, 630090 Novosibirsk, Russia; nuknauer@niikim.ru; 4 Department of Natural Sciences, Novosibirsk State University, 630090 Novosibirsk, Russia; 5 Research Institute of Fundamental and Clinical Immunology, 630099 Novosibirsk, Russia; 6 Clinic for Neurosurgery, Medical Faculty and Heinrich-Heine University Medical Center Düsseldorf, 40225 Düsseldorf, Germany; 7 Molecular and Experimental Surgery, Clinic for General, Visceral, Vascular, and Transplant Surgery, Medical Faculty and University Hospital Magdeburg, 39120 Magdeburg, Germany; ulf.kahlert@med.ovgu.de

**Keywords:** dendrons, phosphorus, supramolecular associates, metallodrugs, copper, gold, tumor stem cells, cytotoxicity, nanomedicine

## Abstract

Dendritic molecules bearing metal complexes in their structure (metallodendrimers and metallodendrons) are considered prospective therapeutic entities. In particular, metallodendrons raise interest as antitumor agents for the treatment of poorly curable or drug-resistant tumors. Herein, we have synthesized amphiphilic triazine-phosphorus dendrons bearing multiple copper (II) or gold (III) complexes on the periphery and a branched hydrophobic fragment at the focal point. Due to their amphiphilic nature, metallodendrons formed single micelles (mean diameter ~9 nm) or multi-micellar aggregates (mean diameter ~60 nm) in a water solution. We have tested the antitumor activity of amphiphilic metallodendrons towards glioblastoma, a malignant brain tumor with a notoriously high level of therapy resistance, as a model disease. The metallodendrons exhibit higher cytotoxic activity towards glioblastoma stem cells (BTSC233, JHH520, NCH644, and SF188 cell lines) and U87 glioblastoma cells (IC50 was 3–6 µM for copper-containing dendron and 11–15 µM for gold-containing dendron) in comparison with temozolomide (IC50 >100 µM)—the clinical standard of care for glioblastoma. Our findings show the potential of metallodendron-based nanoformulations as antitumor entities.

## 1. Introduction

Dendritic molecules, dendrimers and dendrons, are symmetric hyperbranched macromolecules consisting of a core and radially growing branches terminated with multiple functional groups exposed to the surface [1]. Thanks to this feature, along with a precisely defined molecular structure and monodispersity-by-design, dendrimers and dendrons are considered prospective nano-platforms for the development of therapeutic formulations, active both per se or as carriers for bioactive entities [2,3,4,5].

An important class of dendrimers with proven biological activity contains dendritic species bearing metal complexes on the periphery. These dendrimers, referred to as metallodendrimers, consist of a dendritic scaffold (various architecture types reported) decorated with nitrogen-based or *N*-heterocyclic carbene ligands coordinating metal ions. The main field of biological applications of metallodendrimers is antitumor therapy [6,7,8]. Combining the cytostatic/cytotoxic activity of metallodrugs and the multifunctionality of dendrimers, it is possible to create novel formulations to tackle tumors. Metallodendrimers arouse special interest for the treatment of drug-resistant tumors because their mechanism of action generally differs from that of conventional chemodrugs; for instance, poly(alkylidenimine) dendrimers bearing ruthenium (I) complexes exhibit strong activity towards cisplatin-resistant cancer cells [9].

There are numerous examples of metallodendrimers having silicon- and phosphorus-based scaffolds. Carbosilane dendrimers functionalized with monodentate and bidentate ligands, coordinating ruthenium (II) [10,11] and copper (II) [12,13,14,15] complexes, have been reported to suppress the viability of cancer cell lines of different origin in micromolar concentrations. The cytotoxic activity of carbosilane metallodendrimers appears to be stronger than that of polyamidoamine dendrimers bearing metal complexes of similar structure [16,17]. Interestingly, carbosilane metallodendrons can be combined with anti-cancer siRNAs to act both as an oligonucleotide carrier and a metallodrug. This provides a combined effect on tumor cell viability [18,19].

Phosphorus dendrimers bearing copper (II) complexes on the periphery (12 groups for G1, 24 for G2 and 48 for G3) suppress the proliferation of tumor cell lines in a generation-dependent manner (IC50 in the micromolar range), alone [20] or in combination with conventional chemodrugs [21]. Using a gold (III) complex in the form of a [AuCl_2_]^+^[AuCl_4_]^−^ pair, instead of a copper (II) complex, decreased the IC50 down to nanomolar concentrations [22]. The mechanism of the antitumor action of phosphorus metallodendrimers likely involves translocation of the apoptosis-regulating protein Bax into mitochondria, provoking the release of apoptosis induction factors into the cytosol, followed by DNA fragmentation and subsequent cell death [23].

Along with dendrimers, metal-containing dendrons were reported. Dendrons have different functionalities at the focal point (i.e., attached directly to a core) and on the periphery, which can be orthogonally modified. A series of carbosilane dendrons G1–3 bearing a ruthenium (I) complex at the focal point and cationic groups on the periphery has been reported. Dendrons were observed to suppress tumor cell growth and prevent cell adhesion in vitro, as well as reduce xenograft tumor progression in vivo [24].

Another type of dendrons that have been combined with metal complexes are amphiphilic dendrons. This kind of dendrons, bearing hydrophobic fragments at the focal point and hydrophilic groups on the periphery, is known to form supramolecular associates [25]. Therefore, in water solutions (including biological buffers and cultural media), they exist in the form of aggregates, with their size depending on both the dendron structure and medium conditions. Several examples of amphiphilic phosphorus dendrons of generation 1, bearing a linear hydrophobic tail at the focal point and copper (II) or gold (III) complexes on the periphery, have been described. Amphiphilic metallodendrons efficiently induced apoptosis of tumor cells through the translocation of Bax; the IC50 values were in the micromolar range (1–7 µM) [26].

Recently, we have shown that introducing a branched hydrophobic triazine block at the focal point of a dendron, followed by functionalization of a periphery with hydrophilic functional groups, allows us to obtain supramolecular associates with good therapeutic performance [27]. Herein, we expand this methodology to the design of amphiphilic triazine-phosphorus metallodendrons. We report examples bearing copper (II) and gold (III) complexes and assess their antitumor therapeutic performance in a glioblastoma cell model.

## 2. Materials and Methods

### 2.1. Materials

Organic solvents were dried and freshly distilled under argon prior to use. Reagents were obtained from commercial sources and used as received. Bifunctional phosphorus dendron of the AB_5_ topology was obtained as described elsewhere [28].

Solutions of metallodendrons and temozolomide (Sigma-Aldrich, Taufkirchen, Germany) were prepared in DMSO, followed by dilution in MilliQ^®^ deionized water.

### 2.2. Analytical and Spectroscopic Techniques

^1^H, ^13^C{^1^H} and ^31^P{^1^H} NMR spectra were recorded on Bruker AV400PAS and AV300PAS (Bruker, Karlsruhe, Germany) instruments. ^1^H and ^13^C chemical shifts (δ, ppm) were measured relative to residual resonances of solvents. High-resolution mass spectra were recorded using GCT Premier (Waters, Milford, MA, USA) and UPLC Xevo G2 Q TOF (Waters, Milford, MA, USA) mass spectrometers for chemical ionization and electrospray ionization, respectively.

### 2.3. In Vitro Models

We used several cell models of gliomas as an example disease class for unmet clinical need. The in vitro models included a pediatric glioma model (SF188, kindly provided by E. Raabe, Johns Hopkins, Baltimore, MA, USA), a classical glioblastoma culture (U87, kindly provided by A. Weyerbrock, Medical Center Freiburg, Freiburg, Germany) and three stem cell models of glioblastoma: BTSC233 (kindly provided by M.S. Carro, Clinic for Neurosurgery, Medical Center Freiburg, Freiburg, Germany), JHH520 (kindly provided by G. Riggins, Neurosurgery, Johns Hopkins Hospital, Baltimore, MD, USA), NCH644 (kindly provided by C. Herold-Mende, Neurosurgery Freiburg, Clinic for Neurosurgery, Medical Center Heidelberg, Heidelberg, Germany Germany). These models have been recently shown to accurately recapitulate pathophysiological properties of different accepted molecular subtypes of the disease, featuring presentation of prominent DNA mutation profile, DNA methylation pattern according to [29], as well as transcriptomics according to [30]. Our disease modeling technology enables reproducible analysis due to their longitudinal molecular and cellular stability, which is particularly suitable for drug development projects (for details on generation of the cell models and further reference, see [31]).

### 2.4. Synthesis of Amphiphilic Metallodendrons

2,4-dodecylamino-6-chloro-1,3,5-triazine was synthesized and characterized as reported previously [27].

#### 2.4.1. 2,4-dodecylamino-6-(4-hydroxyphenyl)ethylamino-1,3,5-triazine

2,4-didodecylamino-6-chloro-1,3,5-triazine (4.82 g, 10.0 mmol) and tyramine (4.14 g, 30 mmol) were mixed in 150 mL toluene; the reaction mixture was refluxed overnight under argon atmosphere at 110 °C. When the starting triazine derivative was fully consumed, as shown by TLC (30% acetone in hexane), the reaction mixture was cooled down to room temperature; volatiles were removed by rotary evaporator. The solid residue was then dispersed in 150 mL CH_2_Cl_2_ and 20 g of silica gel was added; volatiles were removed first by rotary evaporator then residual solvent was removed under high vacuum. The solid was applied on silica gel column (200 g silica gel in 10% acetone in hexane). The column was washed with 2 L of 10% acetone in hexane, 2 L of 15% acetone in hexane, then product was eluted in 3.5 L 20% acetone in hexane. Fractions containing product were combined, concentrated to viscous oil that turned into white solid after standing overnight at room temperature. Residual traces of solvent were removed under high vacuum. Yield: 5.30 g (95%).

^1^H NMR (300 MHz, CDCl_3_) δ 0.90 (t, J = 6.5 Hz, 6H, H_a_), 1.16–1.41 (m, 36H, H_b_, H_c_), 1.54 (br s, 4H, H_d_), 2.78 (t, *J* = 6.3 Hz, 2H, H_i_), 3.36 (br s, 4H, H_e_), 3.59 (br s, 2H, H_h_), 4.80 (br s, 1H, H_g_), 4.99 (br s, 2H, H_f_), 6.65 (d, *J* = 8.1 Hz, 2H, H_k_), 6.97 (d, *J* = 8.1 Hz, 2H, H_j_). ^13^C{^1^H} NMR (75 MHz, CDCl_3_) δ 14.1, 22.7, 26.9, 28.7–30.4 (m), 31.9, 34.5, 40.7, 115.6, 129.8, 155.4, 163.5–166.6 (m). MS: [M−H]^−^ 581.49; [M] 582.50 amu; [M+H]^+^ 583.51 amu (calcd 582.50 amu).

#### 2.4.2. Aldehyde-Terminated Dendron

2,4-dodecylamino-6-(4-hydroxyphenyl)ethylamino-1,3,5-triazine (870 mg, 1.5 mmol), dendron AB_5_ (1.40 g, 1.8 mmol) and K_2_CO_3_ (310 mg, 2.25 mmol) were mixed in 50 mL of acetonitrile under argon atmosphere and stirred overnight at 75 °C. When the starting triazine derivative was fully consumed, as shown by the shift in aromatic protons’ signals in ^1^H NMR, reaction mixture was filtered hot through the filter paper. The solution was cooled down first to room temperature then kept overnight at −20 °C. Upon cooling down, product precipitated as a white powder that further turned into a viscous oil. Cold supernatant was carefully decanted, the residue was washed with acetonitrile, and the supernatant was decanted again. Remaining solvent was removed under vacuum. Aldehyde-terminated dendron was obtained as slightly yellow viscous oil (1.84 g, 93%).

^1^H NMR (400 MHz, CDCl_3_) δ 0.87 (t, *J* = 6.5 Hz, 6H, H_a_), 1.19–1.39 (m, 36H, H_b_, H_c_), 1.55 (br s, 4H, H_d_), 2.84 (t, *J* = 6.7 Hz, 2H, H_i_), 3.34 (br s, 4H, H_e_), 3.58 (br s, 2H, H_h_), 4.82 (br s, 3H, H_f_, H_g_), 6.91 (d, *J* = 8.0 Hz, 2H, H_k_), 7.06 (d, *J* = 8.2 Hz, 2H, H_j_), 7.08–7.20 (m, 10H, H_l_), 7.71–7.78 (m, 10H, H_m_), 9.94, 9.95 (2 s, 3H and 2H, H_n_). ^13^C{^1^H} NMR (101 MHz, CDCl_3_) δ 14.12, 22.68, 26.97, 29.18–30.12 (m), 31.90, 35.28, 40.71, 120.65 (q, *J* = 1.9 Hz), 121.27 (q, *J* = 2.3 Hz), 129.96, 131.37 (d, *J* = 2.9 Hz), 133.63 (q), 137.05, 148.34–148.46 (m), 154.71 (tq, *J* = 17.3, 5.0, 2.3 Hz), 166.06, 190.33–190.63 (m). ^31^P{^1^H} NMR (162 MHz, CDCl_3_) δ 7.4. MS: [M+H]^+^ 1322.57 amu (calcd 1321.57).

#### 2.4.3. Ligand-Terminated Dendron

Aldehyde-terminated dendron (500 mg, 0.38 mmol) was dissolved in 20 mL THF, then 2-hydrazinopyridine (211 mg, 1.93 mmol) was added. The reaction mixture was stirred overnight at 60 °C. When the aldehyde was fully consumed, as shown by the complete disappearance of aldehyde protons’ signals in ^1^H NMR, the reaction mixture was cooled down to room temperature, and product was precipitated by adding 60 mL of diethyl ether. The supernatant was filtered off, and the residue was washed twice with diethyl ether. Remaining solvent was removed under vacuum. Ligand-terminated dendron was obtained as slightly yellow powder (580 mg, 85%).

^1^H NMR (400 MHz, DMSO-d6) δ 0.80 (m, 6H, H_a_), 1.14 (m, 36H, H_b_, H_c_), 1.42 (br s, 4H, H_d_), 2.80 (br s, 2H, H_i_), 3.16 (br s, 4H, H_e_), 3.37 (2H, H_h_, hidden under the H_2_O signal), 6.31, 6.44 (2 br s, 3H, H_f_, H_g_), 6.72 (t, *J* = 5.9 Hz, 5H, H_p_), 6.87 (d, *J* = 8.1 Hz, 2H, H_k_), 6.93 (m, 4H, H_l_), 7.00 (d, *J* = 8.3 Hz, 6H, H_l_), 7.15 (d, *J* = 7.6 Hz, 2H, H_j_), 7.21 (t, *J* = 7.4 Hz, 5H, H_p_), 7.57 (m, 15H, H_m_, H_p_), 7.99 (s, 5H, H_p_), 8.08, 8.09 (2 s, 5H, H_n_), 10.84, 10.85 (2 s, 5H, H_o_). ^13^C{^1^H} NMR (101 MHz, DMSO-d6) δ 14.37, 22.55, 26.96, 29.04–30.21 (m), 31.76, 106.75, 115.39, 120.84, 121.38, 127.64, 130.22, 133.26 (d, *J* = 3.5 Hz), 137.55, 1138.02, 138.34, 148.13, 148.49, 150.28, 157.45, 165.91. ^31^P{^1^H} NMR (162 MHz, DMSO-d6) δ 8.7. MS: [M+2H]^2+^ 889.93 amu; [M+3H]^3+^ 593.62 amu; [M+4H]^4+^ 445.47 amu; [M+5H]^5+^ 356.57 amu (calcd 1776.83 amu).

#### 2.4.4. Metallodendrons

Metallodendrons were prepared as described in [20,22,26]. Ligand-terminated dendron (53 mg, 0.03 mmol) was dissolved in DMF under argon atmosphere, together with vacuum-dried metal salt. The reaction mixture was stirred at 60 °C overnight. DMF was removed under vacuum, solid residue was washed twice with ethanol and dried under vacuum.

**CuD**: 22.2 mg (0.165 mmol) CuCl_2_ was taken. The metallodendron was obtained as dark-green powder (40 mg, 54%).

**AuD**: 100 mg (0.33 mmol) AuCl_3_ was taken. The metallodendron was obtained as brown powder (60 mg, 40%).

### 2.5. DLS Measurements

CuD and AuD samples were dissolved in DMSO at the concentration 10 mM, then diluted to 50 µM with deionized water. Particle size distribution in dendron solutions was determined in plastic disposable microvolume cells using a Zetasizer Nano S particle analyzer (Malvern, UK). The measurements were made at 25 °C [26,32].

### 2.6. TEM Images

Transmission electron microscopy (TEM) images were obtained using JEM 1400 transmission electron microscope (JEOL, Tokyo Japan) at the accelerating voltage of 100 kV in 50 µL.

### 2.7. Cell Cultivation

BTSC233, JHH520, NCH644, and SF188 cells were cultivated as neurospheres in high-glucose DMEM media without pyruvate (Thermo Fisher, Waltham, MA, USA), containing F12 (3:1) and 1× B27 supplements (both Thermo Fisher, Waltham, MA, USA), 20 ng/mL human EGF, 20 ng/mL human VGF (both Peprotech, Hamburg, Germany), 5 µg/mL heparin (Sigma-Aldrich, Taufkirchen, Germany), 1× penicillin/streptomycin (Sigma-Aldrich, Taufkirchen, Germany) in standard conditions (humidified atmosphere, 37 °C, 5% CO_2_) [33]. U87 cells were cultivated in the following two variants: as a suspension culture (conditions as above) and as an adherent culture in DMEM, supplemented with 10% FBS (Thermo Fisher, Waltham, MA, USA) 1× penicillin/streptomycin.

### 2.8. Cell Viability Assay

Cells were seeded in 96-well flat-bottomed culture plates (10,000 cells in 100 µL total volume), treated by metallodendrons or temozolomide (0.1 µM; 0.3 µM; 1 µM; 3 µM; 10 µM; 30 µM; 60 µM; 100 µM), then incubated for 72 h. Untreated cells were used as a control (non-treated control, NTC) and complete medium was used as a blank control. Each point was obtained from 5 technical repetitions.

To perform MTT assay, 10 µL MTT reagent (3-(4,5-dimethylthiazol-2-yl)-2,5-diphenyl tetrazolium bromide, Sigma Aldrich, Taufkirchen, Germany) was added to every well and mixed thoroughly, plates were incubated ca. 3 h in the dark at RT. Then, 100 µL MTT lysis buffer, containing isopropanol (VWR, Langenfeld, Germany), Triton X (Sigma Aldrich, Taufkirchen, Germany) and HCl (Roth, Karlsruhe, Germany), was added, thoroughly mixed and incubated for 20 min in the dark at RT. Absorbances at 570 nm and 650 nm were read on Paradigm plate reader (Molecular Devices, San Jose, CA, USA). Cell viability was calculated as a ratio of absorbance of treated cells samples to that of non-treated control, then converted into a percentage [34].

Cell viability values were plotted against -lgC, fitted with Boltzmann sigmoidal curve (r^2^ > 0.95), and the -lg(IC50) values were estimated from the fitting data.

### 2.9. Statistical Analysis

We used GraphPad Prism 9 software (San Diego, CA, USA) and Statistica 7.0 (StatSoft, Tulsa, OK, USA) software for data analysis and visualization. The Mann–Whitney criterion was used, and the differences were considered significant if *p* < 0.05.

## 3. Results and Discussion

### 3.1. Synthesis of Amphiphilic Metallodendrons

To prepare the metallodendrons, we have chosen a convergent strategy, where a bifunctional dendritic precursor and a focal point modifier are synthesized separately and then connected via a linker, followed by further functionalization of the dendron periphery. As a dendritic precursor, we have used a bifunctional AB_5_-type phosphorus dendron consisting of a hexafunctional cyclotriphosphazene core bearing five hydroxybenzaldehyde substituents and a chlorine substituent [28].

**Figure 1:**
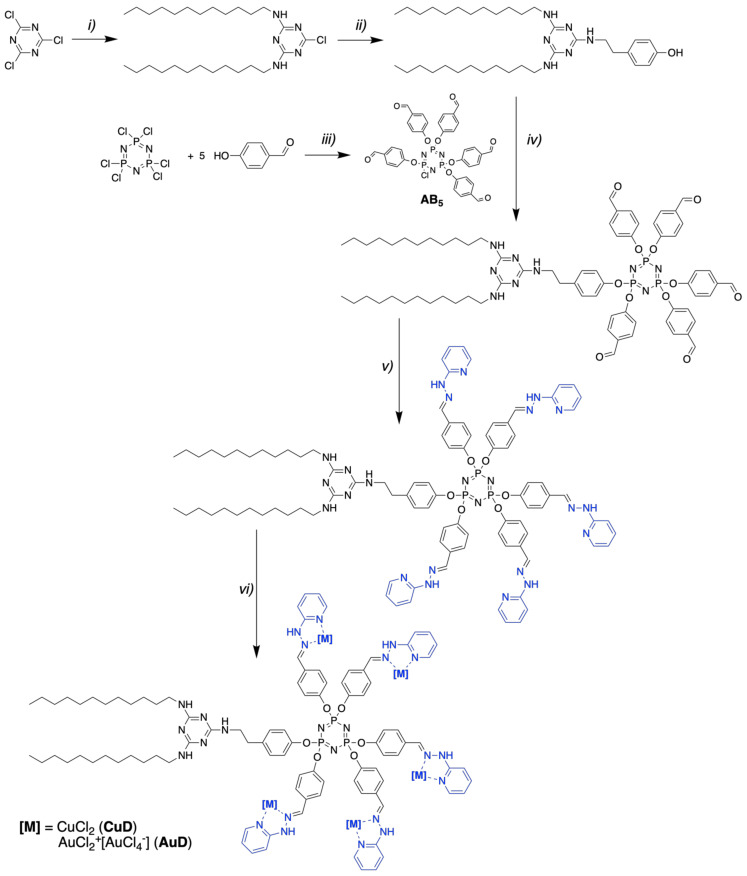
Synthesis of amphiphilic triazine-phosphorus metallodendrons. Conditions: (*i*) *n*-C_12_H_25_NH_2_, CHCl_3_, NaOH (aq.), 90%; (*ii*) tyramine, toluene, 110 °C, 95%; (*iii*) K_2_CO_3_, acetonitrile, 0 °C, 60%; (*iv*) AB_5_, K_2_CO_3_, acetonitrile, 75 °C, 93%; (***v***) 2-hydrazinopyridine, THF, 60 °C, 85%; (*vi*) CuCl_2_ or AuCl_3_, DMF, 60 °C, 54% or 40%.

A hydrophobic triazine modifier was synthesized using a strategy reported earlier [27]. First, cyanuric chloride was modified with two dodecylamine moieties (Figure 1, *i*), then the linker was introduced (Figure 1, *ii*) to graft the hydrophobic modifier to the focal point of a dendron. Herein, we used tyramine as a convenient linker. The grafting of the triazine synthon to the dendron (Figure 1, *iv*) is performed in the presence of an inorganic base (potassium carbonate). Using acetonitrile as a solvent at this stage greatly facilitates the purification of a functionalized dendron, since hydrophobic triazine derivatives are soluble in acetonitrile at >70 °C, but are insoluble at room temperature and below, thus they can be easily separated. To decorate the dendron periphery with ligands for metal coordination (Figure 1, *v*), we have chosen 2-hydrazinopyridine, which is known to form a stable Schiff base upon reaction with benzaldehyde moieties on the dendrimer surface [20]. Being stabilized by the aromatic fragments in the vicinity, the hydrazone formed does not require reduction and makes two nitrogen atoms (imine and pyridine), which are available for coordination. 

It should be noted that the preparation of surface-decorated triazine-phosphorus dendrons described herein is easily scalable. The synthesis of the triazine-modified aldehyde-terminated precursor available for further surface decoration (Section 2.4.2) has been optimized for use at the gram scale. Hydrophobic triazine derivatives are synthesized at the multi-gram scale. The yield per stage is 85+%. To improve the workflow, a tyramine-containing triazine derivative (Section 2.4.1) can be purified by extraction (CHCl_3_: 1M HCl, then washing the organic phase with 0.2M KOH, and with brine), instead of using a silica gel column. A yellowish oil containing traces of toluene is thus obtained, instead of white powder; however, it is satisfactorily pure, as shown by NMR.

At the last stage, copper (II) or gold (III) salts were complexed with ligands on the dendrons’ surface (Figure 1, *vi*). It is worth noting that, unlike copper (II) chloride, gold (III) chloride undergoes disproportioning upon complexation to form an ion pair [LAuCl_2_]^+^ [AuCl_4_]^−^ [22]. Both copper-containing and gold-containing dendrons aggregate into insoluble solids upon storage at room temperature. These findings support previous data stating that pyridinohydrazone is quite a weak ligand for metal coordination, due to its geometry [35]. From the synthetic point of view, this could be a disadvantage; however, the high lability of metal cations in complexes suggests that metallodendrons should be considered as prospective prodrugs for metal-based therapy.

The metallodendrons prepared herein contain multiple hydrophilic metal centers on the periphery and a hydrophobic triazine moiety at the focal point. Thus, we can consider them as the first reported examples of amphiphilic triazine-phosphorus dendrons. These dendritic species expand the family of amphiphilic dendrons containing the main group of elements that already consists of amphiphilic phosphorus [26], carbosilane [36,37,38] and triazine-carbosilane [27] dendrons.

### 3.2. Behavior of Metallodendrons in Aqueous Media

Due to their amphiphilic nature, metallodendrons are expected to exist in solution, not as individual molecules, but rather as supramolecular associates [25]. We have studied their behavior, analyzing the distribution of particles in solutions by means of dynamic light scattering (DLS). It should be noted that as-prepared powders of metallodendrons are poorly soluble in water. However, after being dissolved in DMSO and diluted with water, dendrons form clear stable solutions.

**Figure 2:**
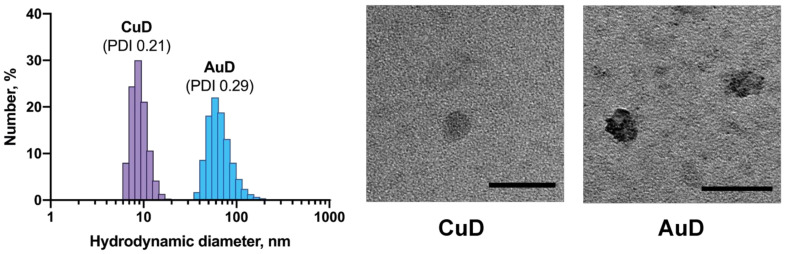
Particle size distribution in CuD and AuD solutions (**left**). TEM images of metallodendrons (**right**). Scale bar is 50 nm.

DLS shows that both CuD and AuD form aggregates of low polydispersity (PDI < 0.3) in water solutions (Figure 2). However, the sizes of these aggregates differ. CuD likely forms single micelles (mean diameter 8.7 nm), whereas AuD is likely associated into bigger multi-micellar aggregates (mean diameter 58.8 nm). Such aggregation of AuD likely originates from inter-molecular interactions between [LAuCl_2_]^+^ [AuCl_4_]^−^ ion pairs on the dendron surface. Importantly, the particle sizes in both samples, though different, are favorable for cellular uptake.

Importantly, being stored as DMSO solutions, metallodendrons generally retain their properties for over 9 months. After dilution with water, AuD forms aggregates with a mean diameter of 45–50 nm (PDI 0.25); CuD forms aggregates with a mean diameter of 20–23 nm (PDI 0.22). It is also worth noting that AuD aggregates keep their morphology, even when stored as a water solution (mean diameter 70 nm, PDI 0.29), whereas CuD samples aggregate upon storage (two subpopulations of particles with mean diameters of 150 nm and 800 nm, PDI > 0.4).

Zeta potential values of the metallodendron aggregates could not be acquired, likely due to their electroactivity. Upon measuring, the metallodendron samples decomposed, which resulted in the appearance of multiple charged forms in the apparent zeta potential profiles (Figure S2).

### 3.3. Cytotoxicity of Metallodendrons towards Glioblastoma Cells and Glioblastoma Stem Cells

As a model for assessing the therapeutic performance of metallodendrons, we have chosen glioblastoma, a highly aggressive malignant brain tumor [39]. Glioblastoma is generally associated with poor prognosis, with a median survival of less than 2 years under a standard-of-care treatment regime [40], due to its invasive growth and therapy resistance. Both features are frequently associated with the activity and residual maintenance of tumor stem-like cell residual upon the resection of tumor bulk. Tumor stem-like cells are also thought to mediate therapy resistance to the only clinical standard-of-care chemotherapy, temozolomide, which is a notorious clinical problem for patients suffering from this disease.

The ability of amphiphilic metallodendrons to suppress tumor cell growth was tested using three lines of glioblastoma stem cells (BTSC233, JHH520, and NCH644), pediatric glioma cells SF188, and U87 glioblastoma cells. U87 is traditionally grown as a monolayer culture in serum containing growth media. To uniform our models, as well as to study the effect of cell morphology on their sensitivity towards metallodendrons, we decided to test both growth variants of this model.

**Figure 3:**
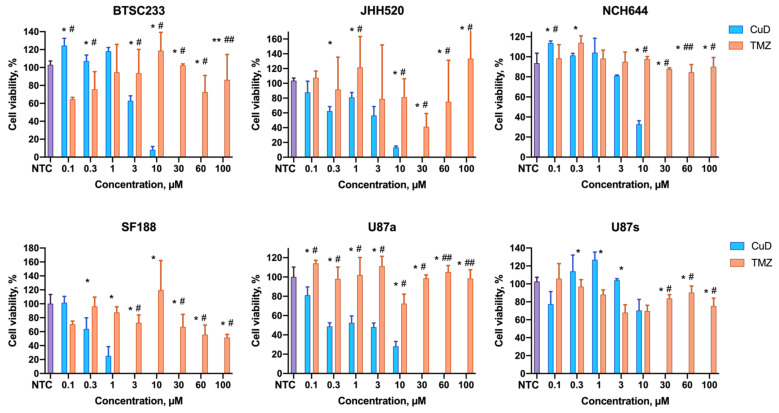
Profiles of the cell viability (MTT assay) after incubation with CuD metallodendrons and temozolomide (TMZ). NTC—non-treated control. Data are presented as mean ± S.D. * *p* < 0.05, ** *p* < 0.01 CuD vs. AuD; # *p* < 0.05, ## *p* < 0.01 CuD vs. TMZ.

**Figure 4:**
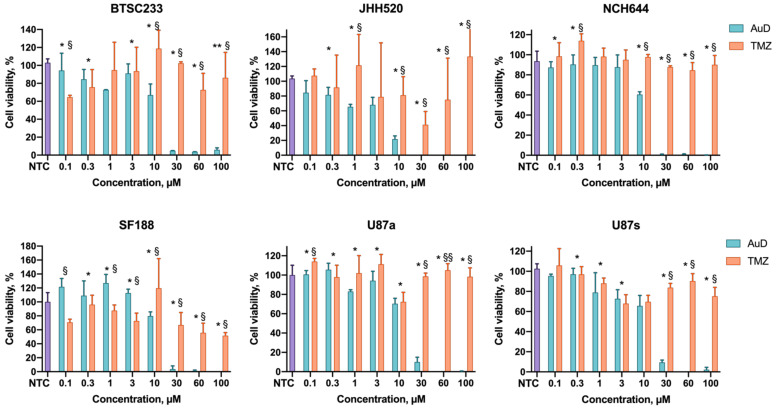
Profiles of the cell viability (MTT assay) after incubation with AuD metallodendrons and temozolomide (TMZ). NTC—non-treated control. Data are presented as mean ± S.D. * *p* < 0.05, ** *p* < 0.01 CuD vs. AuD; § *p* < 0.05, §§ *p* < 0.01 AuD vs. TMZ.

The cell viability after exposure to dendrons for 72 h was assessed by the MTT test. Both dendrons have been found to suppress cell viability in a dose-dependent manner (Figure 3 and Figure 4). Generally, the copper-containing dendron has lower IC50 values than the gold-containing dendron in the cells under study (3–6 µM vs. 11–15 µM, respectively; Table 1). This finding correlates with the differences in the sizes of dendron associates. We may hypothesize that metallodendron micelles have better bioavailability than bigger aggregates.

U87 cells have been shown to be more susceptible to CuD when grown as an adherent culture than in suspension (IC50 4.7 µM vs. 11.1 µM, respectively). These differences are likely connected with alterations in cell membrane properties and uptake mechanisms. In the case of AuD, no significant difference was observed.

Importantly, SF188 cells appeared to be remarkably sensitive to the copper-containing metallodendrimers. The IC50 of CuD has been found to be as low as 0.34 µM, whereas the IC50 of the standard-of-care drug temozolomide was >100 µM. This finding is especially important when considering that the SF188 cell line represents pediatric glioma, a rare disease, which is suspected to be particularly aggressive. Further validation of nanodrugs as particularly effective therapy options for malignant pediatric brain tumors, despite enormous research efforts and unmet clinical need with devastating outcomes for families, is ongoing.

It should be noted that upon the treatment of cells with temozolomide, which is the only drug accepted as standard of care in the Western world to treat glioblastoma, 50% suppression of the cell viability has not been reached in the whole range of concentrations used (0.1–100 µM). Therefore, we could not determine IC50 values for temozolomide from the data obtained. For SF188 cells, we roughly estimated the IC50 value from the fitting parameters (Table 1). Thus, the cells under study appeared to be less sensitive to the standard chemotherapy, unlike metallodendrons.

We nevertheless acknowledge a technical limitation of our study. Although we applied advanced three-dimensional stem-cell-derived disease modeling of the disease we studied, resembling various hallmark molecular features of the original tumors [31], we did not address the aspect of the brain barrier penetration potential of our new drug class. Although patients suffering from glioblastoma have a leaking blood–brain barrier, which can even be detected in clinical imaging [41], it would be desirable to test the brain bioavailability of our metallodrugs using xenograft mice carrying the tumors of our used cell model. Moreover, human alternatives using induced pluripotent stem cells have emerged [42]. However, we believe that they do not fully recapitulate the shear stress and perfusion properties of an organism. Therefore, in vivo assessments of the pharmacokinetics and tissue penetration of the presented metallodendrons are ongoing in our labs.

## 4. Conclusions

Herein, we have synthesized the first examples of a new dendritic species—amphiphilic triazine-phosphorus dendrons. The examples obtained bear multiple metal complexes (copper (II) or gold (III)) on the periphery of the cyclotriphosphazene core-based dendron, as well as a branched hydrophobic fragment at the focal point. Due to their amphiphilic nature, the metallodendrons formed supramolecular associates in water solutions.

However, the methodology that we have described is compatible with other functional groups that can be introduced onto the dendron surface, such as charged moieties, biomimetics, targeting fragments, functional polymers, etc. Considering that triazine chemistry is also flexible and permits various types of nucleophiles to be introduced into the structure, the synthetic strategy we propose permits libraries of functional dendrons bearing diverse moieties on the periphery and at the focal point to be obtained.

We show promising proof-of-concept data on the antitumor activity of metallodendrons towards cancer stem cells, which are comparatively poorly affected by the standard-of-care clinical drug temozolomide, suggest that the use of metallodendrons should be expanded to combat tumor drug resistance. Meanwhile, the physicochemical properties of nanoformulations (storage stability, polydispersity of supramolecular associates formed, and their behavior in biological media) should be taken into account and improved, if necessary. This can be performed either by biophysical approaches, i.e., choosing appropriate medium conditions for storage and use, or by the chemical synthesis of dendritic species with improved physicochemical and pharmacological characteristics.

We believe that amphiphilic triazine-phosphorus dendrons are useful scaffolds for the design of functional dendritic molecules. The metallodendron species we report here are promising entities for nanomedicine-based therapy of poorly curable tumors, with indications that they may possess the highest potential for the unmet clinical need of malignant pediatric brain tumors.

## Figures and Tables

**Table 1:** IC50 values of metallodendrons and temozolomide (TMZ).

Cell Line	IC50, µM		
	CuD	AuD	TMZ
BTSC233	3.2 ± 0.3	11.5 ± 2.9	n/a ^1^
JHH520	4.8 ± 1.1	6.4 ± 2.0	n/a
NCH644	5.9 ± 0.8	11.3 ± 2.8	n/a
SF188	0.34 ± 0.21	12.3 ± 2.5	~125 ^2^
U87 (adherent)	4.7 ± 3.0	15.0 ± 3.6	n/a
U87 (suspension)	11.1 ± 3.8	14.9 ± 2.8	n/a

^1^ IC50 could not be determined; ^2^ value predicted from data fitting.

## Data Availability

The raw data supporting the conclusions of this article will be made available by the authors upon reasonable request.

## Supplementary Materials

The following are available online at https://www.mdpi.com/article/10.3390/pharmaceutics14020393/s1, Figure S1: NMR spectra of new compounds synthesized; DLS profiles of metallodendrons’ aggregates upon storage; zeta potential profiles of metallodendrons’ aggregates. Figure S2: DLS profiles of CuD and AuD metallodendrons stored for >9 months. Figure S3: Zeta potential profiles of 50 µM water solutions of CuD and AuD metallodendrons.
