# Supplementary material for: Amphiphilic Triazine-Phosphorus Metallodendrons Possessing Anti-Cancer Stem Cell Activity
Source: Pharmaceutics. 2022 Feb 10;14(2):393. doi: 10.3390/pharmaceutics14020393 (PMC8880151; doi:10.3390/pharmaceutics14020393)
Supplement: Supplementary file 1 [file pharmaceutics-14-00393-s001.zip › pharmaceutics-1523516-supplementary.pdf]

pharmaceutics

MDPI

Supplementary Materials: Amphiphilic Triazine-Phosphorus Metallodendrons Possessing Anti-Cancer Stem Cell Activity

Evgeny K. Apartsin, Nadezhda Knauer, Ulf Dietrich Kahlert and Anne-Marie Caminade

S1. NMR Spectra

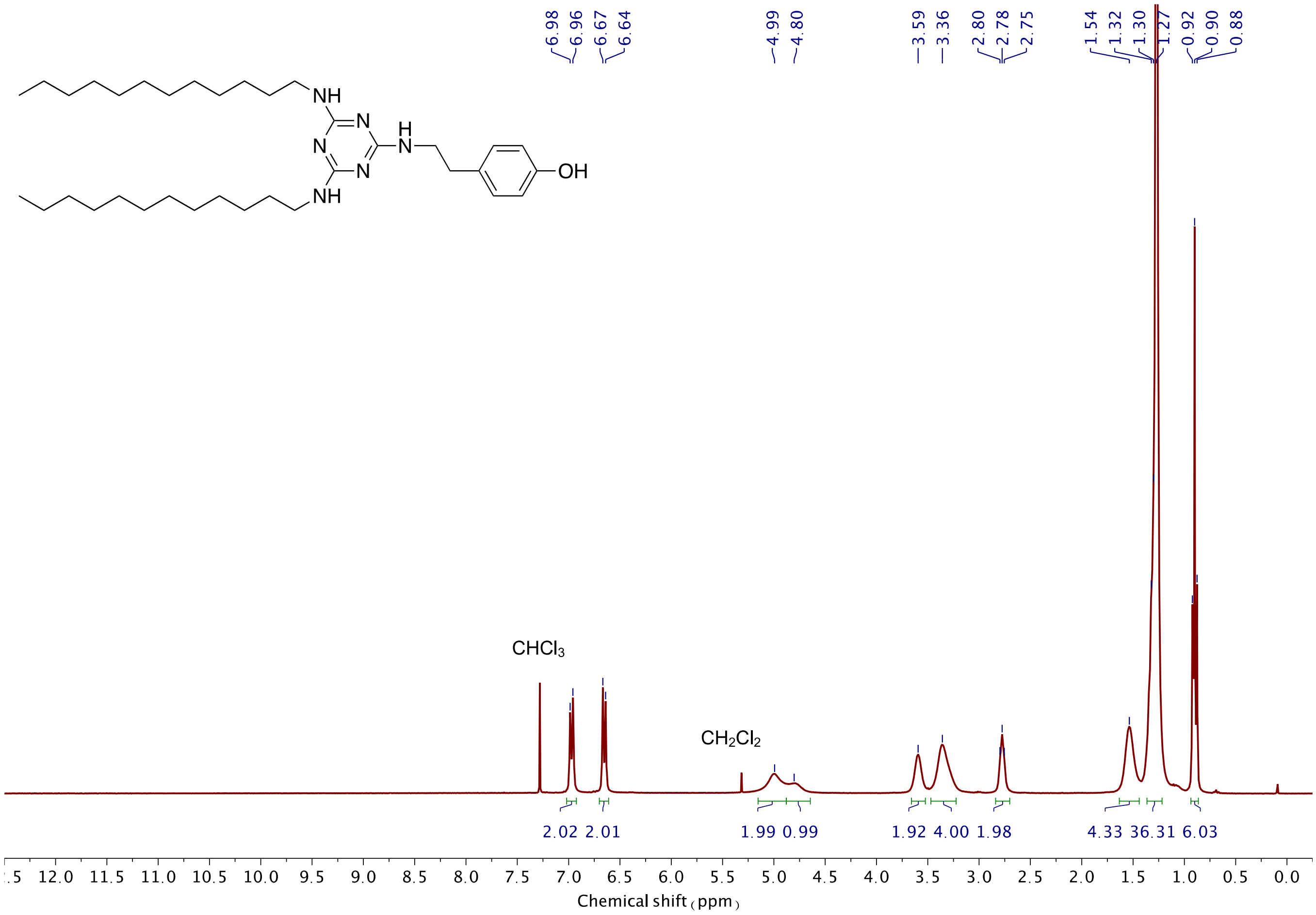

^{1}H NMR (300 MHz, $CDCl_3$).

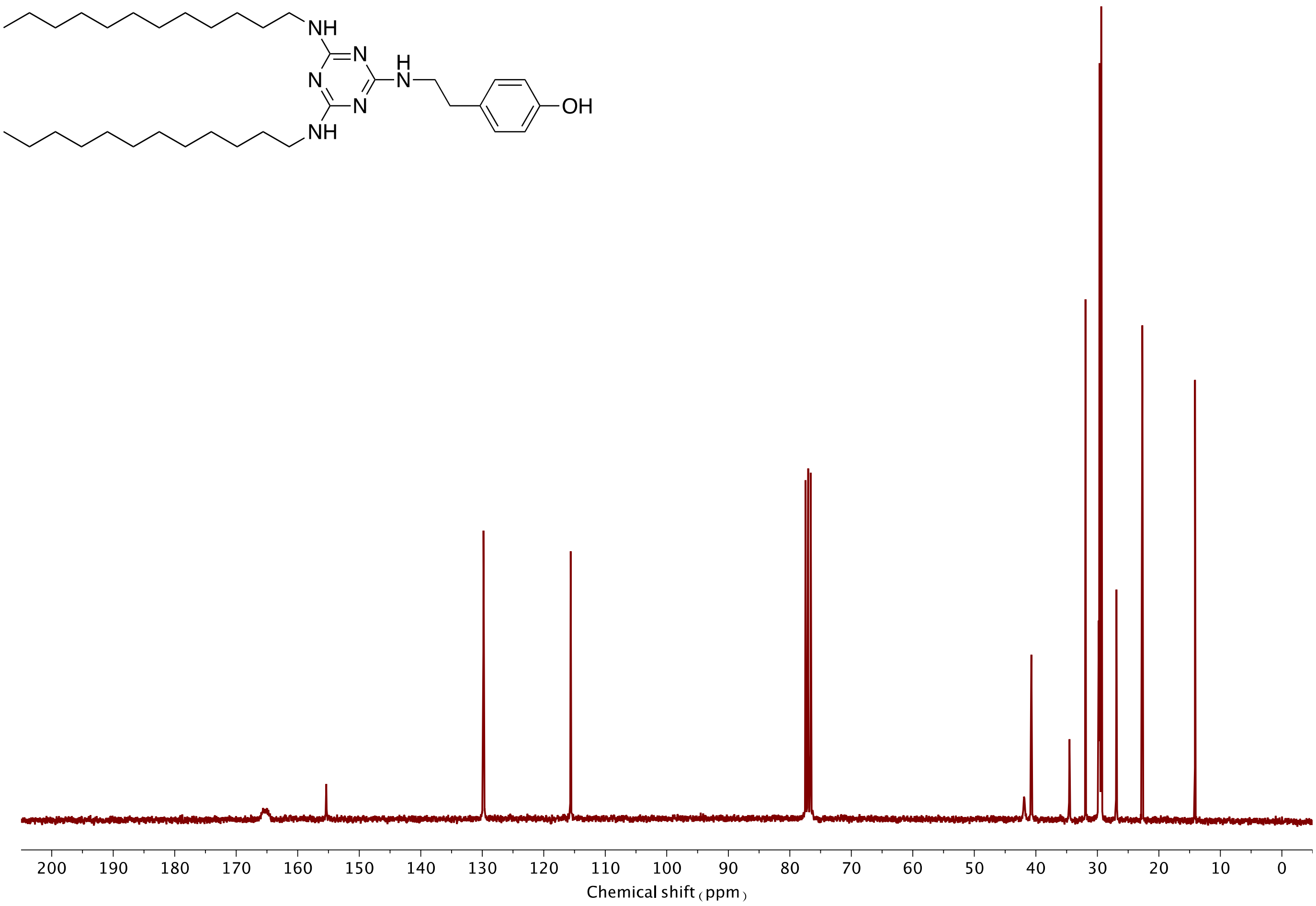

$^{13}C\{^{1}H\}$ NMR (75 MHz, $CDCl_3$).

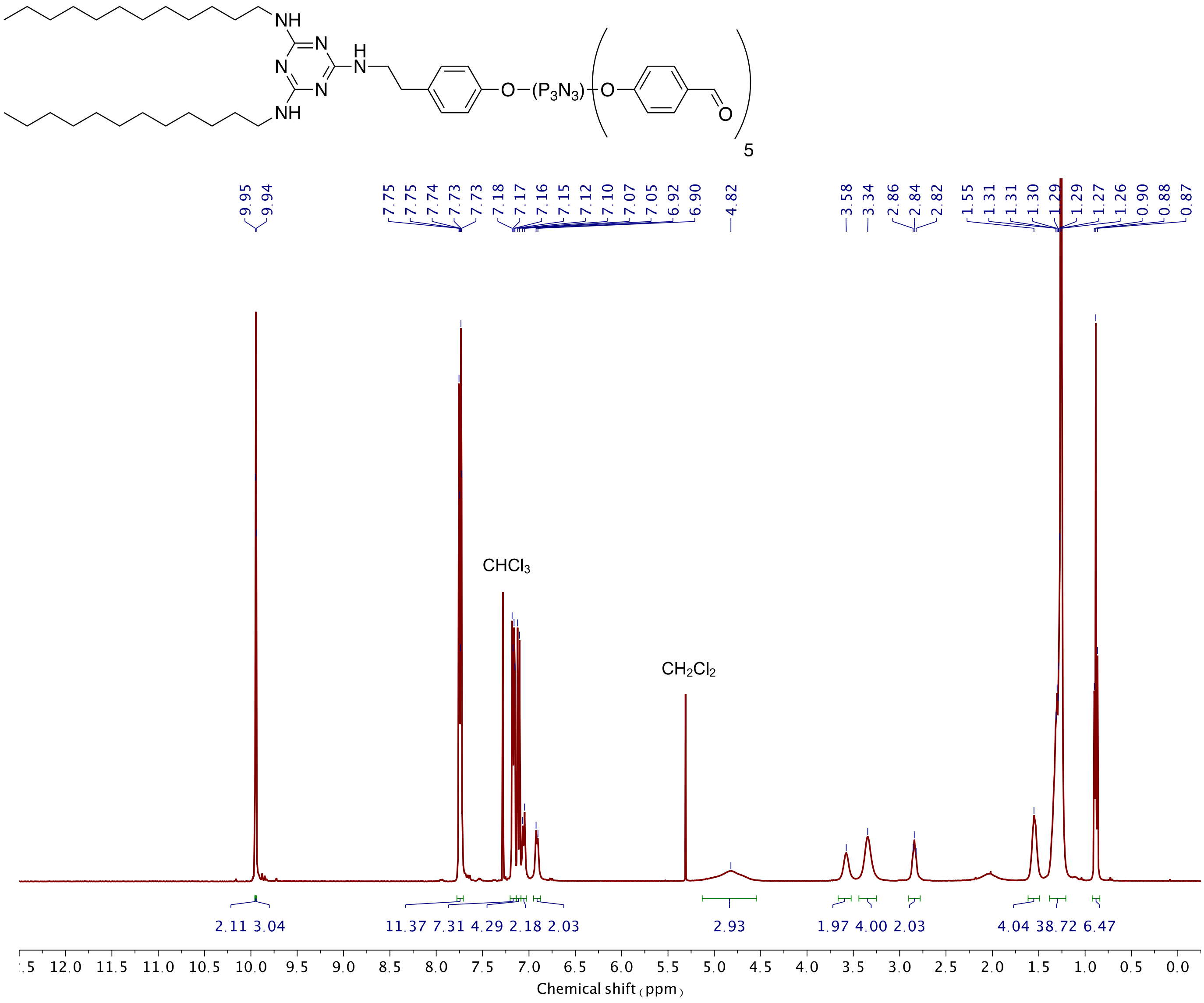

^{1}H NMR (400 MHz, $CDCl_3$).

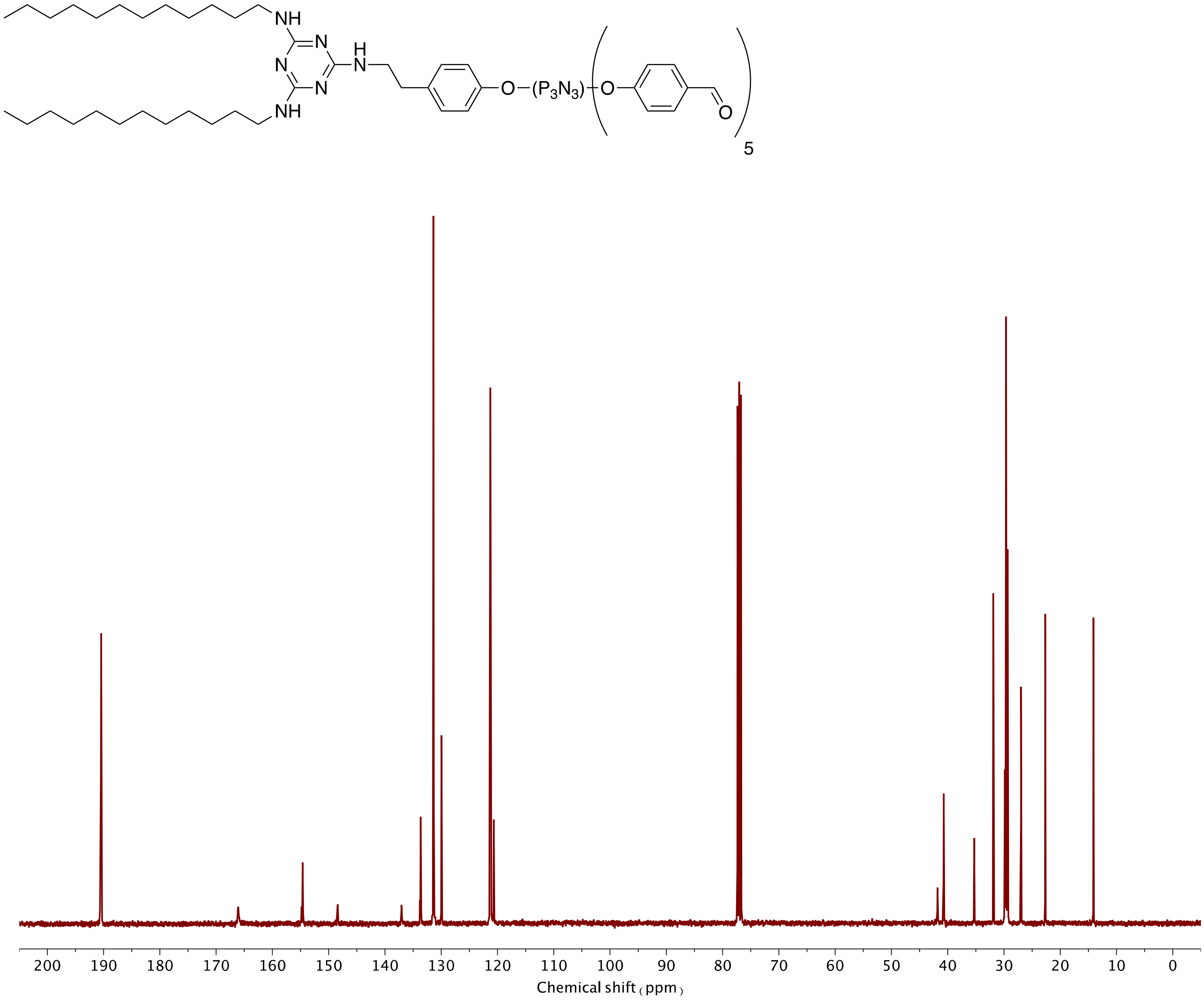

$^{13}C\{^{1}H\}$ NMR (101 MHz, $CDCl_3$).

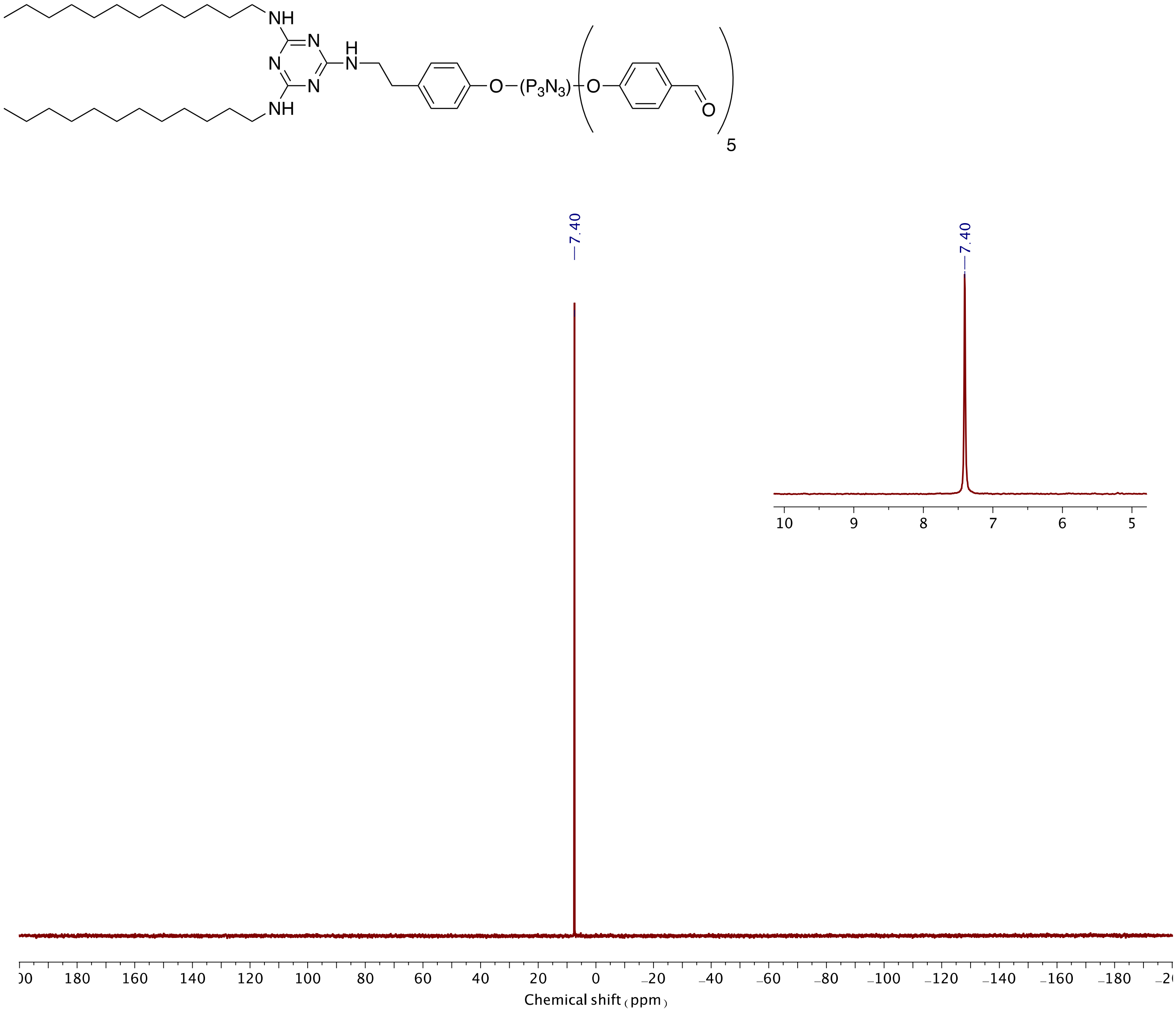

$^{31}P\{^{1}H\}$ NMR (162 MHz, $CDCl_3$).

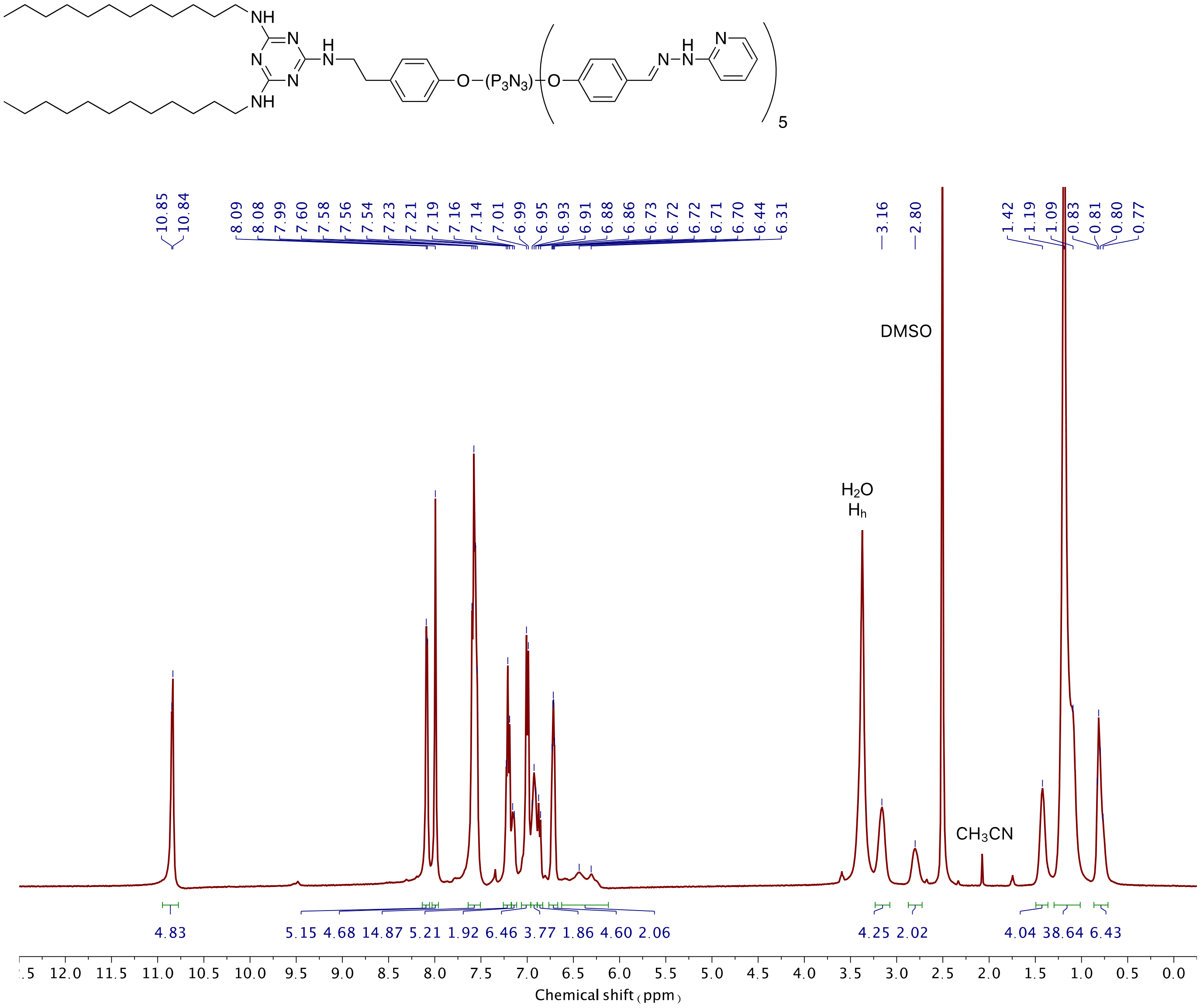

^{1}H NMR (400 MHz, DMSO-d6).

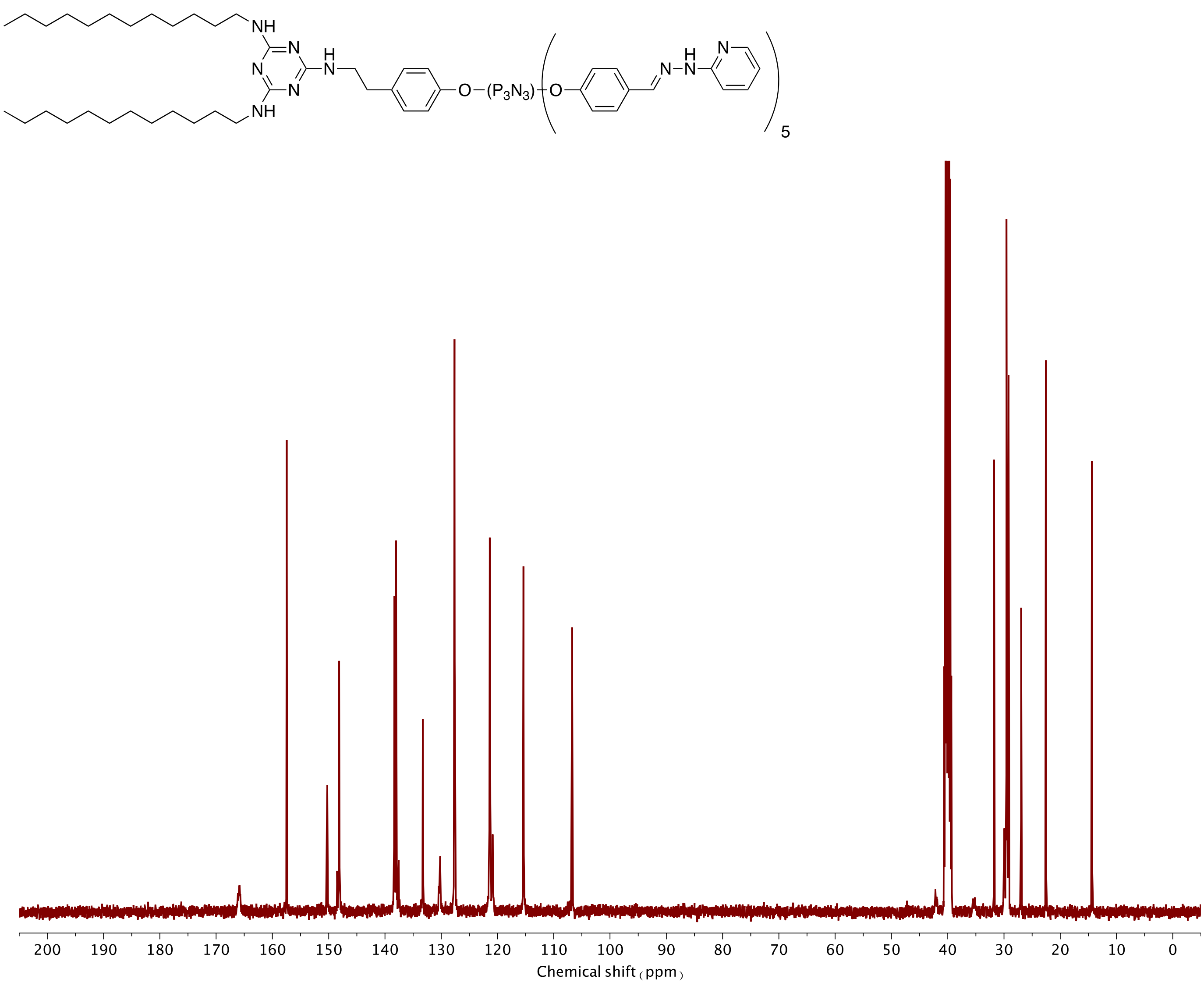

$^{13}C\{^1H\}$ NMR (101 MHz, DMSO-d6).

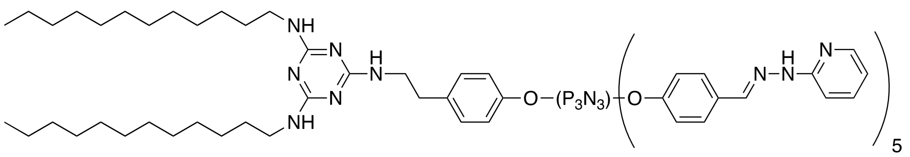

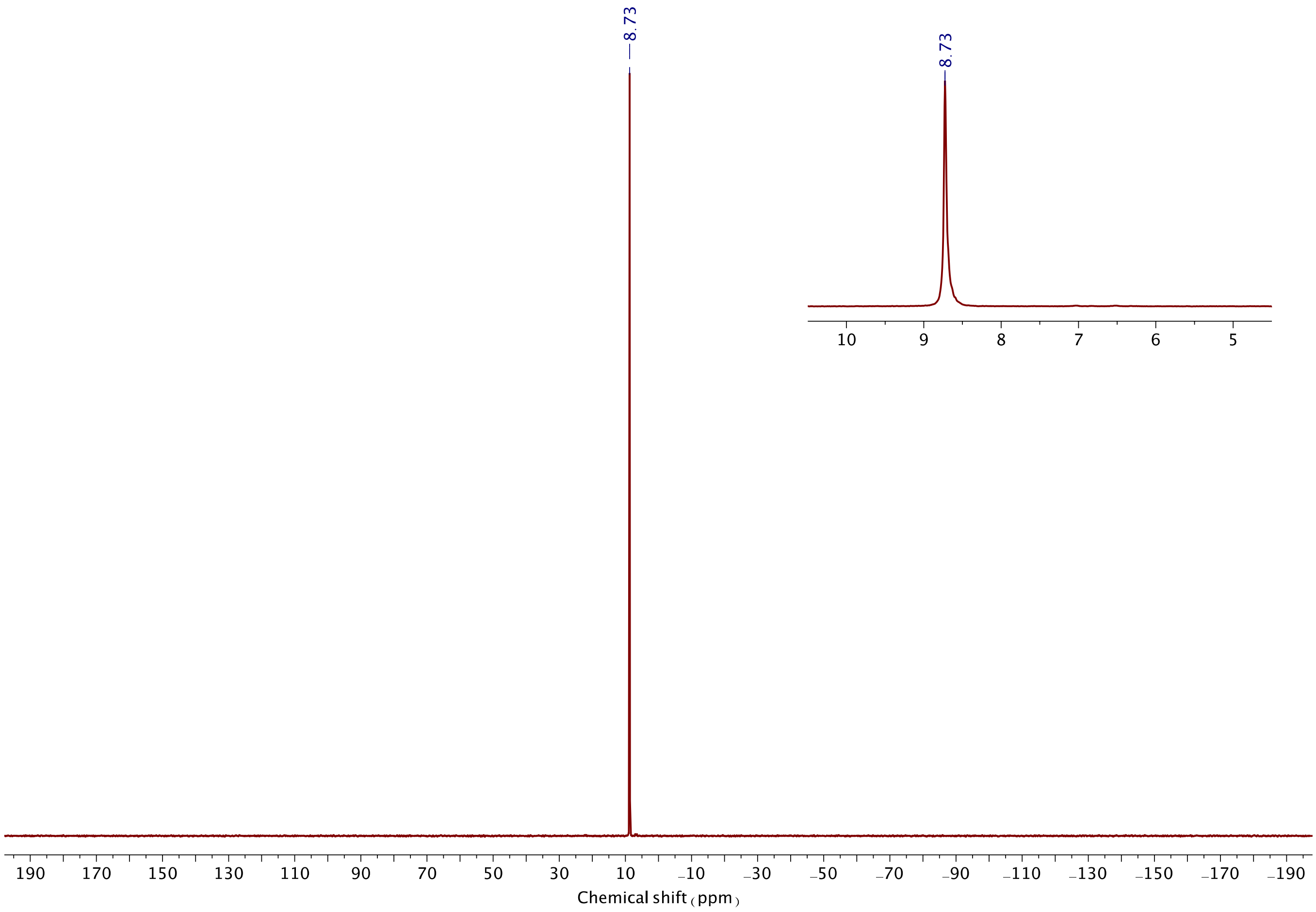

$^{31}P\{^{1}H\}$ NMR (162 MHz, DMSO-d6).

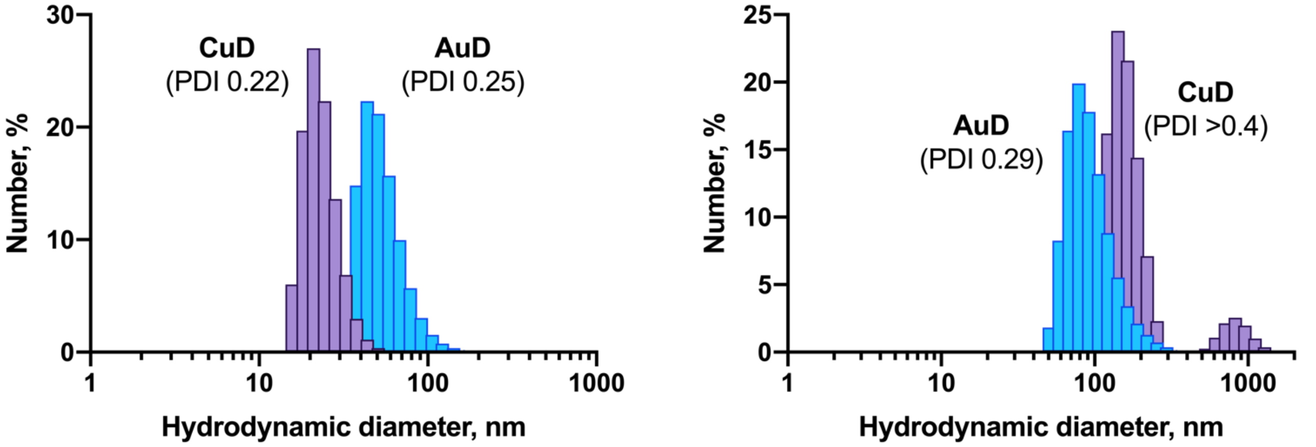

Figure S2. DLS profiles of CuD and AuD metallodendrons stored for >9 months as a DMSO solution and diluted to 50 μM before measurement **(left)**. DLS profiles of CuD and AuD metallodendrons stored for >9 months as 50 μM solution in water (**right**).

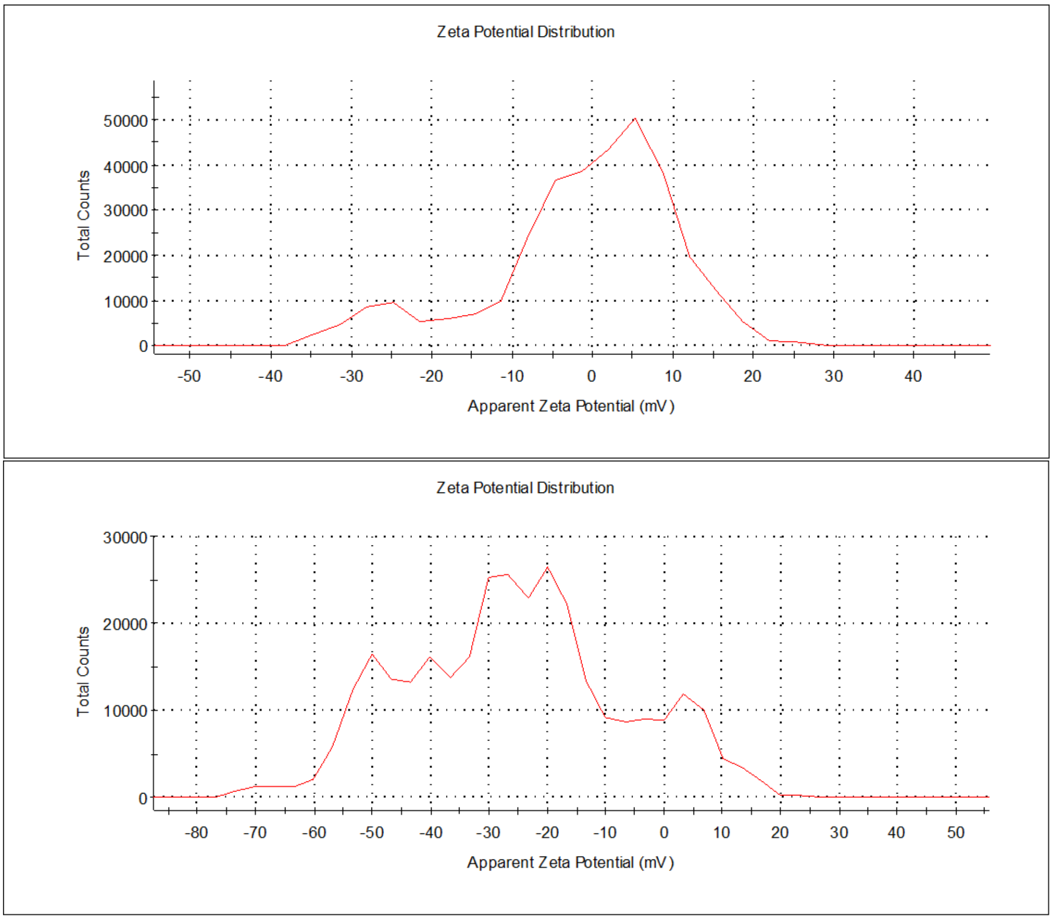

Figure S3. Zeta potential profiles of 50 μM water solutions of CuD (**top**) and AuD (**bottom**) metallodendrons.
